# Assessing Aquatic Baseline Toxicity of Plastic-Associated Chemicals: Development and Validation of the Target Plastic Model

**DOI:** 10.1021/acs.jcim.4c00574

**Published:** 2024-08-09

**Authors:** Deedar Nabi, Aaron J. Beck, Eric P. Achterberg

**Affiliations:** † GEOMAR Helmholtz Centre for Ocean Research Kiel Wischhofstr. 1-3, 24148 Kiel, Germany; ‡ Institute of Environmental Science and Engineering (IESE), School of Civil and Environmental Engineering (SCEE), National University of Sciences and Technology (NUST), H-12, Islamabad, Pakistan

## Abstract

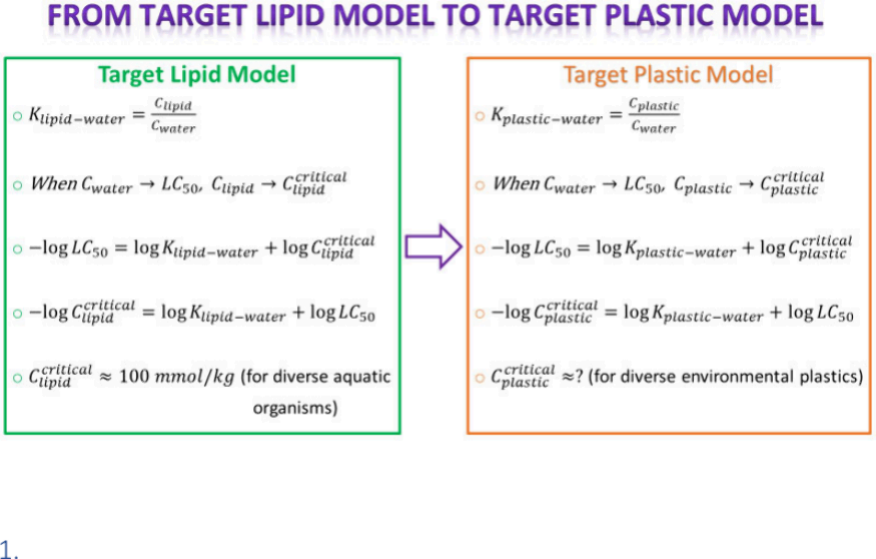

We developed a Target Plastic Model (TPM) to estimate the critical plastic burden of organic toxicants in five types of plastics, namely, polydimethylsiloxane (PDMS), polyoxymethylene (POM), polyacrylate (PA), low-density polyethylene (LDPE), and polyurethane ester (PU), following the Target Lipid Model (TLM) framework. By substituting the lipid–water partition coefficient in the TLM with plastic–water partition coefficients to create TPM, we demonstrated that the biomimetic nature of these plastic phases allows for the calculation of critical plastic burdens of toxicants, similar to the notion of critical lipid burdens in TLM. Following this approach, the critical plastic burdens of baseline (*n* = 115), less-inert (*n* = 73), and reactive (*n* = 75) toxicants ranged from 0.17 to 51.33, 0.04 to 26.62, and 1.00 × 10^–6^ to 6.78 × 10^–4^ mmol/kg of plastic, respectively. Our study showed that PDMS, PA, POM, PE, and PU are similar to biomembranes in mimicking the passive exchange of chemicals with the water phase. Using the TPM, median lethal concentration (LC_50_) values for fish exposed to baseline toxicants were predicted, and the results agreed with experimental values, with RMSE ranging from 0.311 to 0.538 log unit. Similarly, for the same data set of baseline toxicants, other widely used models, including the TLM (RMSE: 0.32–0.34), ECOSAR (RMSE: 0.35), and the Abraham Solvation Model (ASM; RMSE: 0.31), demonstrated comparable agreement between experimental and predicted values. For less inert chemicals, predictions were within a factor of 5 of experimental values. Comparatively, ASM and ECOSAR showed predictions within a factor of 2 and 3, respectively. The TLM based on phospholipid had predictions within a factor of 3 and octanol within a factor of 4, indicating that the TPM’s performance for less inert chemicals is comparable to these established models. Unlike these methods, the TPM requires only the knowledge of plastic bound concentration for a given plastic phase to calculate baseline toxic units, bypassing the need for extensive LC_50_ and plastic–water partition coefficient data, which are often limited for emerging chemicals. Taken together, the TPM can provide valuable insights into the toxicities of chemicals associated with environmental plastic phases, assisting in selecting the best polymeric phase for passive sampling and designing better passive dosing techniques for toxicity experiments.

## 1 Introduction

Plastic has undoubtedly brought numerous benefits to modern society. Nevertheless, the plastic revolution has also had significant impacts on global ecosystems. Plastic pollution has become ubiquitous, infiltrating our air,^1,2^ water,^3^ soil,^4^ biota,^5^ and even our food,^6^ as well as human blood^7^ and fetal fluids.^8^ Disentangling the complexity of plastic pollution is a daunting task, but at its simplest, environmental issues associated from plastic arise from three key factors: the particulate nature of plastic itself,^9^ which includes macro-, micro-, and nanoplastics; the harmful microorganisms that can harbor on plastic debris;^10,11^ and the chemicals associated with plastic.^12^

Chemicals associated with plastic can be categorized as native chemicals, which are the additives added during manufacturing,^13^ and non-native chemicals that plastic materials accumulate once released into the environment.^14^ As a result, plastic plays a crucial role in the transportation of both native and non-native chemicals in the environment. These chemicals can potentially leach from the plastic phase into various environmental waters, such as freshwater, marine water, and soil pore water.^13−15^

The leaching of chemicals from plastic is influenced by several factors, including the properties of the chemicals, the properties of the plastic materials, water chemistry, and fluid dynamics. The properties of chemicals that affect the uptake and release of chemicals from plastic materials are intermolecular interactions such as polarity, polarizability, hydrogen bonding, and dispersion interactions.^16^ The properties of plastic materials include interaction terms that correspond to the intermolecular interactions of molecules mentioned above.^16^ Furthermore, factors such as crystallinity and the weathering age of plastic materials also play a role in the uptake and release of chemicals, both at equilibrium and under kinetic conditions.^17^

Chemical exchange between water and the rubbery (amorphous) fraction of plastic is primarily governed by partitioning (absorption) processes, whereas the crystalline fraction of plastic generally follows adsorption processes.^18^ The impact of weathering age on the exchange of chemicals between water and plastic materials has not been extensively studied.^19^ Water chemistry, such as pH, dissolved organic matter, and salinity, can influence the exchange of chemicals between plastics and water, particularly under kinetic conditions.^19^ Finally, flow regimes, including turbulent, laminar, and stagnant conditions, can affect the thickness of the diffusion boundary layer between the water phase and plastic phase, which, in turn, has an impact on the kinetic exchange of chemicals between the two phases. For instance, under turbulent conditions, the thickness of the aqueous boundary layer is thinner compared to stagnant conditions, resulting in faster kinetics.^20^

The amorphous or rubbery portion of plastic phases mimics organisms in terms of the passive uptake of chemicals.^21^ These plastic phases only take up the freely dissolved fraction, which also defines the bioavailable fraction, of the total concentration of chemicals.^22^ The total concentration, also termed nominal concentration, consists of the freely dissolved fraction as well as fractions that are associated with particulate matter and dissolved organic matter. Plastic materials selectively take up the truly dissolved fraction of contaminants due to the structure of polymers, characterized by small free volumes resulting from nonideal packing of polymer chains. These free volumes, typically angstroms in size,^23^ allow only small, freely dissolved molecules to diffuse and equilibrate within the polymer matrix. Larger colloidal or nanoparticle-bound chemicals are sterically hindered from entering the confined spaces. Studies have shown that materials such as polydimethylsiloxane (PDMS), polyethylene (PE), polyoxymethylene (POM), polyacrylate (PA), ethylene–vinyl acetate (EVA), and polyvinyl chloride (PVC) uptake the freely dissolved fraction from water containing particulate and dissolved organic matter.^24−27^ It is the freely dissolved fraction of the chemical concentration that truly defines its passive exposure to organisms.^28^ However, dietary intake and biomagnification can also play important roles in determining the body burden of an organism, particularly within aquatic food webs. Taken together, plastic phases such as PDMS, POM, PA, EVA, high-density polyethylene (HDPE), and low-density polyethylene (LDPE) can help to isolate and quantify this fraction, which can be a valuable tool for studying the behavior of chemicals in aquatic environments and their potential impacts on organisms.^24^

As a result, organic environmental chemists have begun to use plastic phases as passive sampling^29^ and dosing devices.^30^ Polymeric phases such as PDMS, POM, PA, EVA, HDPE, and LDPE are used to monitor the truly dissolved concentration of organic pollutants in air, water,^29^ sediments, biotic media, and humans.^31^ Passive dosing methods utilize the same polymeric phases as passive sampling and provide precise control over exposure concentrations of hydrophobic chemicals in laboratory experiments involving multiple phases.^30^

Biomembranes play a crucial role as a barrier against xenobiotics in organisms, serving as a nonspecific, baseline site of toxicity.^32^ These membranes are primarily composed of phospholipids,^33^ and the partition coefficients of these chemicals between the phospholipids and external media, such as water (*K*_lipid–water_), drive their passive uptake through the biomembrane (eq 1).
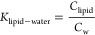
1

When the truly dissolved concentration of a chemical *C*_w_ is equal to or exceeds the median lethal concentration (LC_50_) in the external medium, the chemical can reach a critical level on the biomembrane that can cause toxic injury to the membrane’s functioning. This concentration on the biomembrane is called the critical membrane burden or critical lipid burden (*C*_lipid_^crit^).

Hence, *C*_w_ → LC_50_, *C*_lipid_ → *C*_lipid_^crit^, eq 1, can be rearranged in the following logarithmic form:
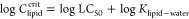
2

For many inert chemicals that interact with biomembranes nonspecifically, it has been observed that the critical membrane burden is fairly constant, with a median value of around 100 mmol/kg (= −1 mol/kg in log units) for many aquatic species, such as fish.^34^ In this case, eq 2 can be rewritten as
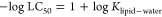
3

A linear regression plot of −log LC_50_ against log *K*_lipid__–water_ should result in an intercept and slope close to unity. The Target Lipid Model (TLM) is based on this concept and assumes that the critical burden is primarily driven by the partition coefficient between the phospholipids and the water phase.^35^

In this study, we borrowed the TLM framework to formulate the Target Plastic Model (TPM) as follows. We assume that the lipid in the TLM can be substituted with plastic, given its biomimetic nature, to create the TPM. In this case, the partition coefficient (*K*_plastic–water_) is a ratio of concentration of a chemical in the plastic phase (*C*_plastic_) to that in the water phase (*C*_w_) at equilibrium.
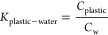
4

Assuming that, when *C*_w_ → LC_50_, *C*_plastic_ → *C*_plastic_^crit^, eq 4 becomes
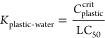
5

Eq 5 can be rearranged in the following logarithmic form:
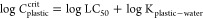
6

The critical plastic burden (log *C*_plastic_^crit^) of chemicals can be calculated if the log LC_50_ and log *K*_plastic–water_ data of chemicals are available.

In this study, our hypothesis is that our new target plastic model can be parametrized with the log *C*_plastic_^crit^, which is expected to remain relatively constant for a particular type of plastic, similar to its counterpart, *C*_lipid_^crit^. This parametrization will enable reliable predictions of LC_50_ with comparable performance to other widely used models, including the target lipid model. We propose three tests to evaluate this hypothesis:

1. First, we will explore the extent of similarity between the biotic phases involved in determining chemical toxicity and various types of plastic. We will evaluate the similarity between these phases based on the intermolecular interactions they experience while interacting with the chemicals, such as polarity, polarizability, hydrogen bonding, and dispersion interactions. This similarity will be inspected by dimensionality analysis, pairwise correlation, and linear regression analyses between log *K*_plastic–water_ and log *K*_biotic-phase-water_ (partition coefficient between the biotic phases and water).
2. Second, we will estimate log *C*_plastic_^crit^ values for various plastic phases using eq 6 and then calculate the median of the resulting distribution. We will also estimate log *C*_plastic_^crit^ as the intercept obtained by linear regression of log *K*_plastic–water_ against–log LC_50_ values for a diverse set of chemicals. The variance in *C*_plastic_^crit^ compared to *C*_lipid_^crit^ will be examined to perform a plastic sensitivity distribution (PSD) analysis, similar to the concept of species sensitivity distribution (SSD) analysis.
3. Third, we will compare the predicted LC_50_ values obtained by putting the *C*_plastic_^crit^ value into the TPM with the experimental values and predictions of other widely used models.

Successful testing of the hypothesis would provide a viable means for environmental scientists to establish a direct link between the quantification of native and non-native pollutants on plastic phases in the environment and chemical risk assessment. This would also enhance the ability of scientists to design their passive sampling and dosing experiments in laboratory and field settings.

## 2 Material and Method

Experimental acute toxicity data of diverse chemicals reported for the fish were taken from the compilations available in the literature.^36^ The compilation comprises experimental LC_50_ values for 949 chemicals. Experimental Abraham solute descriptors were obtained for these chemicals from the freely available online UFZ-LSER database,^37^ resulting in complete sets of experimental Abraham solute parameters for 587 chemicals. Due to the lack of experimental Abraham solute parameters, the remaining 488 chemicals were not considered for further analysis. Based on their toxic mode of action, the final set of 587 chemicals were categorized into three groups: baseline toxicants or nonpolar narcotics, less-inert toxicants or polar narcotics, and reactive toxicants. To evaluate and validate the TPM, the following five sets were created from these three groups.

The first set, called the Nonpolar Narcotics or Baseline Evaluation Set, comprised 115 chemicals that are known to act via a baseline or nonpolar narcotic mode of toxic action.^38^ These chemicals belong to chemical families such as alkanes, alcohols, ketones, ethers, alkyl benzenes, and their chlorinated derivatives. The critical lipid and plastic burden values were derived using this set. This set is available as Table S1 in the Supporting Information (SI).

The second set, called the Baseline Validation Set (Table S2), contains 132 chemicals that were predicted to follow the baseline mode of toxic action according to the baseline model (BL). These chemicals belong to diverse chemical families such as alkyl halides, alkenes, fluoroalcohol, chloroalcohol, diols, triols, alcohols, ethers, esters, carboxylic acids, amines, amides, carbamates, triazine, sulfides, disulfides, sulfoxides, organophosphates, aromatic aldehydes, phthalates, halogenated phenols, nitrobenzenes, and pyridines. While some of these chemicals belong to chemical classes that are typically considered out of the domain of baseline toxicity, many of them are moderately to highly hydrophobic in nature and have been found to act via baseline modes of toxic action, despite containing polar functional groups, as reported in the literature.^36,39^ The critical plastic and lipid burden values derived using the Baseline Evaluation Set were used to predict the LC_50_ values for this set, which were done to independently validate the TPM.

The third set, called the Polar Narcotics or Less-Inert Evaluation Set (Table S3), comprised 73 chemicals that are known to exert slightly higher toxicity than the baseline compounds via polar narcotic mode of toxic action.^36^ These chemicals belong to classes such as phenols, halogenated phenols, and anilines. The critical plastic and lipid burdens of polar narcotic chemicals were obtained by analyzing this set.

The fourth set, called the Less-Inert Validation Set (Table S4), consisted of 128 chemicals, for which the critical lipid and plastic burden values obtained using the Polar Narcotic Evaluation Set were used to predict the LC_50_ values. The predictions were then compared to the experimental LC_50_ values to validate the TPM for polar narcotic chemicals. The selection of these chemicals was based on literature and findings^36,38,40−42^ that classify them as having a known polar mode of toxic action. Additionally, some chemicals were included because the Less-Inert Model (LIM) predictions of their LC_50_ values deviated from experimental values by less than 1 log unit, in line with the methodology described by Wang et al.^36^

The fifth set, called the Reactive Chemical Set (Table S5), comprised 75 chemicals and belonged to chemical families such as aldehydes, benzaldehydes, halogenated benzaldehydes, α,β-unsaturated esters, diamines, dinitrobenzenes, and their hydroxy derivatives. Reactive chemicals are known to covalently react with proteins or DNA in organisms and exert toxicities far above those predicted by nonpolar and polar narcosis.

The partition coefficients of 587 chemicals were estimated for various biotic phases (phospholipid, storage lipid,^43^ muscle protein,^44^ and blood protein^45^), technical solvents (octanol^46^ and triolein^47^), and plastic phases (PDMS,^48^ PA,^49^ POM,^50^ and PE^16^) using Abraham Solvation Model (ASM) equations, with the input of experimental Abraham solute parameters. The ASM was only available for the polyurethane ester (PU)–air system and not for the PU–water system. Therefore, partition coefficients for the PU–water values were obtained by using a thermodynamic cycle between ASM estimated partition coefficients for the PU–air^51^ and air–water^52^ systems. These data are available in Tables S1–S5 in the Supporting Information.

The ASM equations for five other plastic types, namely polypropylene (PP), polystyrene (PS), polyvinyl chloride (PVC), ultrahigh molecular weight polyethylene (UHMWPE), and high-density polyethylene (HDPE), were not available to estimate the partition coefficient between water phases and these plastic phases. Although some experimental plastic–water partition coefficient data for these plastic types were available for small sets of chemicals,^53^ the experimental toxicity data for these chemicals were very sparse (Table S6). The Target Plastic Model was also evaluated for these plastic phases. However, the evaluations for these plastic types could not be considered to be as reliable as those for the following five plastic types: PDMS, PA, POM, LDPE, and PU phases. This is mainly due to the limited experimental partitioning and toxicity data available for the former plastic types. In this study, these plastic phases (PP, PS, PVC, UHMWPE, and HDPE) are collectively termed “other plastics”.

The critical burdens for three groups of chemicals, namely, nonpolar narcotics, polar narcotics, and reactive toxicants, were estimated using three different methods. As a starting point, the first method involved assuming a critical burden of 100 mmol/kg of lipid or plastic for all groups, a value that is widely supported in the literature for lipids.^34^ This method will be termed the “100 mmol method” in the subsequent text. In the second method, which will be referred to as the median method henceforth, the critical burden of each chemical group was calculated by using eq 2 and eq 6 to respectively calculate the critical lipid and critical plastic burden for each chemical in the Baseline Evaluation Set, Polar Narcotic Evaluation Set, and Reactive Chemical Set. The median of the distribution of these burden values was used to represent the critical lipid and plastic burdens for each group of chemicals. In the third method, hereafter referred to as the intercept method, −log LC_50_ values were linearly regressed against log *K*_plastic–water_ (in the case of plastics) and log *K*_lipid__–water_ (in the case of lipid) for chemicals in each group. The intercept obtained from this linear regression respectively represents the critical lipid and plastic burdens according to eq 2 and eq 6. The slopes obtained in these cases indicate the strengths or sensitivity of plastic phases or lipid phases in deriving the toxicity of chemicals, which is similar to species sensitivity analysis toward toxicants using the target lipid model.^35^ We evaluated these three methods of estimating the critical burdens to determine which one produces the best results for predicting toxicities.

To evaluate and validate the TPM, the critical plastic burden values obtained using the three methods described above, and the partition coefficients for plastic/water were inserted into eq 6 to predict the LC_50_ of chemicals in the five chemical sets. The resulting predictions were then compared with experimental values. The same procedure was repeated for TLM (eq 2), allowing for a comparative analysis. In addition to experimental values, predictions of other widely used models were also used to compare the performance of the TPM. To estimate LC_50_ values for the chemicals, the ASM calibrated for several fish species^54^ was used. Additionally, the US-EPA’s ECOSAR module^55^ was utilized, which categorizes the chemicals into ECOSAR classes based on their structure and functional groups. This categorization helps to identify whether the chemical follows a baseline mode of toxic action or a specific mode of action. Moreover, two previously published models,^36^ BL and LIM, were used to predict LC_50_ values. These models are based on linear regression of −log LC_50_ against −log *K*_ow_ and were calibrated using baseline chemicals and less-inert chemicals.

In this study, diverse data sets were used, consisting of chemicals with varying chemical structures, including branched/unbranched/cyclic aliphatic and aromatic compounds, and several different types of functional groups. These chemicals exhibit a broad range of toxicities, hydrophobicities, and intermolecular interactions. Baseline Evaluation and Validation Sets covered a range of −log LC_50_ spanning 6 and 7 orders of magnitude, respectively. The hydrophobicity of these sets ranged over 6 and 8 orders of magnitude, respectively. Polar Narcotic Evaluation and Validation Sets encompassed toxicities with a range spanning more than 9 and 7 orders of magnitude, respectively, and their hydrophobicities covered more than 6 orders of magnitude. The Reactive Chemical Set consisted of chemicals with toxicities ranging from 1.79 to 7.34 log units and octanol–water partition coefficients ranging from −1.88 to 5.05 log units. The wide range of Abraham solute descriptors for the chemicals in these sets indicates their diversity in terms of intermolecular interactions.

## 3 Results and Discussion

### 3.1 Biomimetic Nature of Plastic Phases

How closely the plastic phases mimic organisms depends on the extent to which the intermolecular interactions governing log *K*_plastic–water_ of chemicals resemble those controlling the log *K*_biotic-phase-water_. This can be investigated using approaches based on dimensionality analysis, pairwise correlation, and linear regression between log *K*_plastic–water_ and log *K*_biotic-phase-water_. The results of these analyses are presented below.

**Figure 1:**
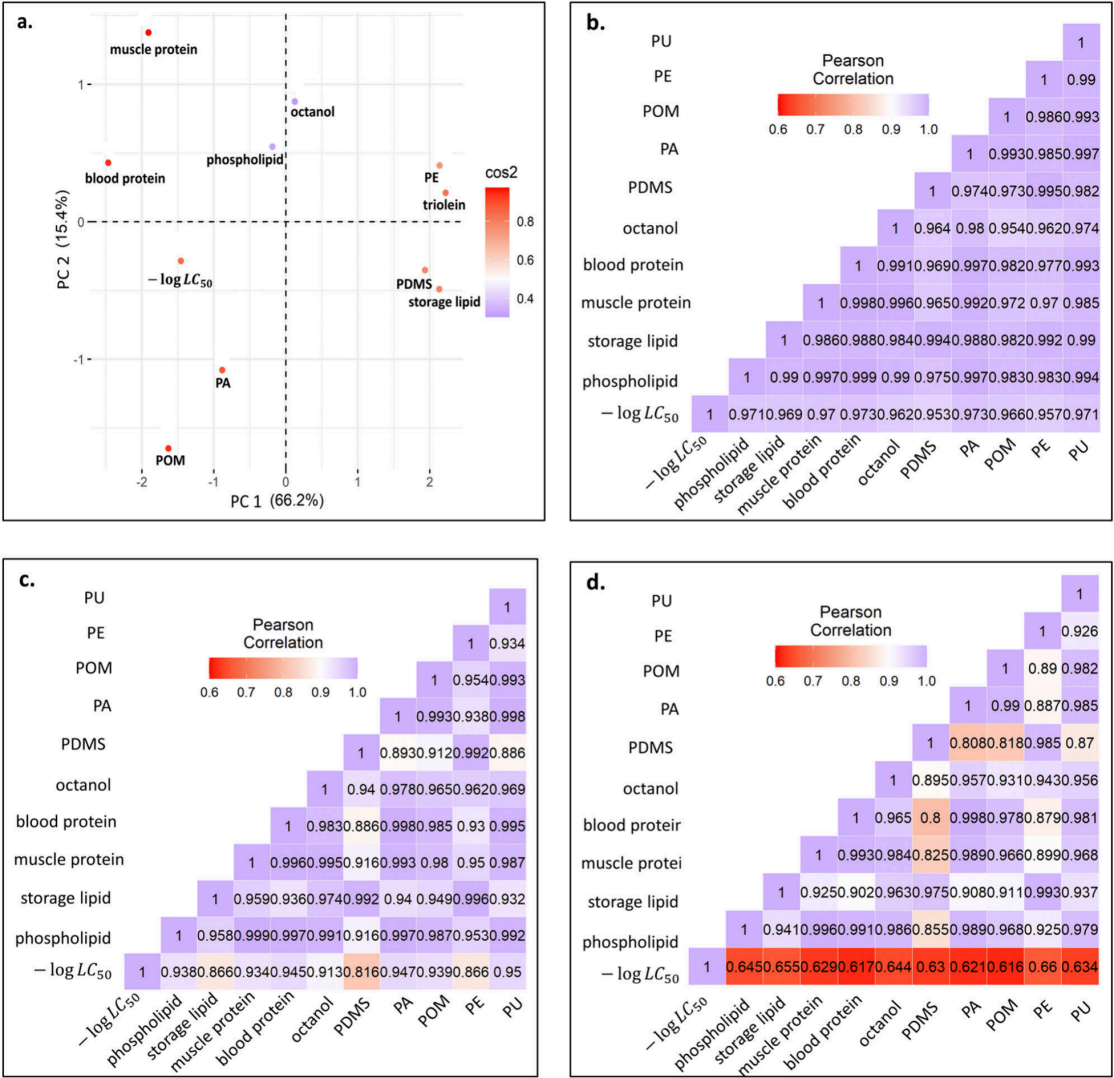
Overlap in information between the biotic and plastic phases in terms of intermolecular interactions and partition coefficients. Panel a illustrates a cluster biplot obtained by performing principal component analysis on the system coefficients of ASM equations for biotic, technical solvents, plastic phases, and the toxicity end point (LC_50_). PC 1 and PC 2, representing principal components 1 and 2, respectively, collectively account for 81.6% of the information, and square cosine (cos2) reflects the quality of phase representation on the biplot. Panels b–d showcase the Pearson’s pairwise correlation between the biotic and plastic phases for baseline (*n* = 115), less-inert (*n* = 73), and reactive (*n* = 75) groups of chemicals.

Dimensionality analyses were performed to assess similarities among the biotic phases (phospholipid, storage lipid, muscle protein, and blood protein), technical solvents (octanol and triolein), plastic phases (PDMS, PA, POM, and LDPE), and toxicity end point (LC_50_). The published system coefficients of the ASM equations available for these phases were used for the analysis. The first two dimensions obtained from the PCA test on the standardized system coefficients of ASM equations for toxicity, biotic phases, technical solvents, and plastic polymers represent 81.6% of the total information encoded in the ASM equations (Figure 1a). The ASM equations for these phases are calibrated using experimental data sets that are diverse in terms of both intermolecular interactions and chemical structures, making the similarity among these phases more representative than if they were based solely on the evaluation sets.

The Euclidean distance found between −log LC_50_ and log *K*_PA-water_ is the lowest compared to distances between −log LC_50_ and other phases observed on the PCA biplot representing the first two dimensions. The next closed plastic phase to −log LC_50_ is the POM polymer. This indicates that log *K*_PA-water_ and log *K*_POM-water_ are the closest allies of −log LC_50_ in terms of intermolecular interactions such as polarizability, polarity, hydrogen bonding interaction, and dispersion forces. The PDMS and PE phases tend to respectively cluster with the storage lipid and triolein phase, indicating the chemical similarities between these phases, suggesting that the PDMS and PE phases can be considered appropriate phases for estimating bioaccumulation. Octanol depicts good proximity to the phospholipid, which corroborates the previous success of TLM where octanol was taken as a proxy for phospholipid. Protein phases were found to be loners in this analysis.

The biomimetic nature of plastic phases can further be discerned by the linear relationship between the toxicity, plastic materials, and biotic phases. For the three groups of chemicals, log *K*_plastic–water_ for the five types of plastics (PDMS, PA, POM, LDPE, and PU) exhibited a strong pairwise correlation with the partition coefficients for the biotic phases (phospholipid, storage lipid, muscle protein and blood proteins), and technical solvent (octanol; Figure 1b–d). The degree of correlation of −log LC_50_ with the partition coefficients for biotic phases were in the same neighborhood as was found with the log *K*_p-w_. This supports the notion that the uptake of chemicals by these plastic phases mimics the uptake by the biotic phases. The degree of correlation between −log LC_50_ and log *K*_plastic–water_ for the five types of plastics was found to be very strong (*r* > 0.95) for baseline toxicants (Figure 1b), strong (*r* > 0.82) for less-inert toxicants (Figure 1c), and moderate (*r* > 0.62) for reactive toxicants (Figure 1d). This corroborates the fact that the toxicity is mainly driven by the partitioning properties of baseline toxicants, whereas the contribution of partitioning in describing the toxicity for reactive chemicals decreases significantly.

The regression statistics such as *R*^2^ and RMSE observed for the linear relationships between −log LC_50_ and log *K*_plastic–water_ were similar to the ones found for the relationships between log LC_50_ and log *K*_biotic-phase-water_ (Table 1). For these linear relationships, the intercept represents the −log *C*_plastic_^crit^ (mol/kg of plastic), and the slope represents the partitioning sensitivity of the plastic compared to the biomembrane. For the baseline toxicants (*n* = 115), a linear regression of log LC_50_ against the log *K*_plastic–water_ for the five types of plastics resulted in equations with intercept (1.43–3.80) and slope (0.78–0.971) with *R*^2^ and RMSE in ranges of 0.947–0.908 and 0.307–0.406, respectively (Table 1). The intercept and slope obtained by linear regression of log LC_50_ against the log *K*_phospholipid-water_ and log *K*_ow_ were 1.07 and 1.13 and 0.96 and 0.90, respectively. The linear relationship is similar to the TLMs for various species reported in the literature.^35^ Phospholipid is considered to be a more accurate phase to use for calculations of the critical lipid burden of narcotic chemicals. As evident by the comparisons of the fitting coefficients of the equations obtained by regressing log LC_50_ against the log *K*_storage-lipid-water_ and against log *K*_phospholipid-water_, the critical lipid burden is overestimated by a factor of 3.6, if the storage lipid, instead of the phospholipid, is considered as the target lipid. However, the phospholipid shows stronger partitioning sensitivity toward the baseline chemicals than the storage lipid. In many cases, the total lipid pool is used to normalize the toxicity end points. For instance, taking into account the total lipid pool of an organism, rather than solely the phospholipid portion, may result in an overestimation of the critical burden by a factor of 2 for baseline toxicants.

**Table 1:** Regression Coefficients for the Equation^a^ −log LC_50_ = −log *C*_biotic-or-plastic-phase_^crit^ + *m* log *K*_biotic-or-plastic-phase-water_, Based on Data Fitting from the Baseline, Less-Inert, and Reactive Sets

group	log *K*_biotic-or-plastic-phase-water_	–log *C*_biotic-or-plastic-phase_^crit^	*m*	RMSE	*R*^2^	*n*
baseline toxicants	phospholipid–water	1.071 ± 0.069	0.962 ± 0.022	0.322	0.942	115
	storage lipid–water	1.627 ± 0.060	0.762 ± 0.018	0.331	0.939	115
	pooled lipid–water	1.369 ± 0.061	0.854 ± 0.019	0.314	0.945	115
	muscle protein–water	1.800 ± 0.055	1.121 ± 0.026	0.325	0.941	115
	serum protein–water	0.881 ± 0.070	1.162 ± 0.026	0.310	0.946	115
	octanol–water	1.127 ± 0.078	0.900 ± 0.024	0.367	0.925	115
	PDMS–water	1.933 ± 0.066	0.780 ± 0.023	0.406	0.908	115
	PA–water	1.403 ± 0.059	0.954 ± 0.021	0.307	0.947	115
	POM–water	1.774 ± 0.059	0.971 ± 0.024	0.344	0.934	115
	PE–water	2.034 ± 0.061	0.808 ± 0.023	0.387	0.916	115
	polyurethane–water	3.803 ± 0.030	0.828 ± 0.019	0.319	0.943	115
less-inert toxicants	phospholipid–water	2.153 ± 0.111	0.737 ± 0.032	0.292	0.880	73
	storage lipid–water	3.144 ± 0.109	0.552 ± 0.038	0.420	0.751	73
	pooled lipid–water	2.677 ± 0.111	0.646 ± 0.035	0.353	0.824	73
	muscle protein–water	2.767 ± 0.089	0.806 ± 0.037	0.302	0.872	73
	serum protein–water	2.013 ± 0.109	0.857 ± 0.035	0.275	0.894	73
	octanol–water	2.529 ± 0.115	0.628 ± 0.033	0.344	0.833	73
	PDMS–water	3.660 ± 0.095	0.575 ± 0.048	0.486	0.666	73
	PA–water	2.317 ± 0.096	0.739 ± 0.030	0.270	0.897	73
	POM–water	2.574 ± 0.093	0.806 ± 0.035	0.289	0.882	73
	PE–water	3.352 ± 0.096	0.662 ± 0.045	0.422	0.749	73
	polyurethane–water	4.396 ± 0.032	0.674 ± 0.026	0.264	0.902	73
reactive toxicants	phospholipid–water	3.623 ± 0.1_50_	0.609 ± 0.083	0.890	0.423	75
	storage lipid–water	4.098 ± 0.113	0.439 ± 0.061	0.899	0.412	75
	pooled lipid–water	3.887 ± 0.125	0.522 ± 0.071	0.888	0.426	75
	muscle protein–water	4.064 ± 0.116	0.694 ± 0.099	0.904	0.404	75
	serum protein–water	3.528 ± 0.167	0.696 ± 0.101	0.912	0.394	75
	octanol–water	3.723 ± 0.144	0.568 ± 0.081	0.905	0.404	75
	PDMS–water	4.374 ± 0.107	0.412 ± 0.062	0.923	0.380	75
	PA–water	3.816 ± 0.135	0.565 ± 0.081	0.907	0.401	75
	POM–water	3.995 ± 0.122	0.546 ± 0.079	0.912	0.395	75
	PE–water	4.339 ± 0.103	0.484 ± 0.065	0.886	0.429	75
	polyurethane–water	4.396 ± 0.032	0.674 ± 0.026	0.907	0.401	75

a The term −log *C*_biotic-or-plastic-phase_^crit^ denotes the critical burden of chemicals on the biotic or plastic phase, obtained as the intercept of a plot of log LC_50_ against the partition coefficient for the biotic or plastic phase (log *K*_biotic-or-plastic-phase-water_). The slope of the equation is represented by *m*.

Among plastic phases, PA demonstrated the best fit with *R*^2^ = 0.947 and RMSE = 0.307 log unit for baseline toxicants. The slopes observed for the relationships of log LC_50_ against the log *K*_plastic–water_ of each PA and POM phases were in close agreement with slopes observed for TLMs based on phospholipid–water and octanol–water. This indicates that the partitioning property of baseline toxicants for the PA and POM materials is similar to that for the target lipid. The critical plastic burden—as indicated by the intercepts—of baseline toxicants for the plastic phases was lower than the ones found for the target lipids.

In the case of less-inert toxicants (*n* = 73), the fit statistics for the linear relationships log LC_50_ and log *K*_biotic-phases-water_ and between −log LC_50_ and log *K*_plastic–water_ are satisfactory, although not as good as those found for baseline toxicants. For the five types of plastics, values of RMSE and *R*^^2^^ ranged from 0.26 to 0.49 log units and from 0.67 to 0.902, respectively, with PU showing the best fits and PDMS showing the least good fits. For the five types of biotic phases, values of RMSE and *R*^2^ were in the ranges of 0.28 to 0.42 log units and 0.67 to 0.902, respectively. Regression of log LC_50_ against log *K*_ow_ depicted *R*^2^ = 0.83 and RMSE = 0.344 log unit. The relatively inferior statistics for the less-inert toxicants indicate that factors other than partitioning are important to account for the toxicity variability for this category of toxicants. The response sensitivities (slopes) are lower for less-inert chemicals than those found for baseline toxicants, and the critical burdens are lower for less-inert chemicals than those for baseline toxicants, which is expected as less-inert chemicals are more toxic than the baseline toxicants.

The regression of log LC_50_ against log *K*_biotic-phases-water_ and log *K*_plastic–water_ for reactive toxicants (*n* = 75) yielded unsatisfactory fit statistics, with *R*^2^ values ranging from 0.380 to 0.429 and RMSE values spanning a range of 0.886–0.923 log units. These results suggest that the reactive mode of toxic action for these chemicals is not well modeled by the partitioning models. Reactive toxicants are known to react covalently with cellular components, such as proteins and DNA, and form adducts that alter their structure and function, leading to toxicity. As a result, TPM and TLM, which have limitations of capturing such covalent interactions, are expected to be ineffective for such chemicals.

Overall, these results support previous findings that plastic phases behave similarly to biotic phases in terms of exchanging baseline toxicants with the aqueous phases. This similarity remains adequate for less-inert chemicals but cannot be reliably established for reactive toxicants. Therefore, the biomimetic properties of plastic can be utilized to formulate TPM for baseline and less-inert toxicants.

### 3.2 Critical Plastic Burden vis-à-vis Critical Lipid Burden

**Figure 2:**
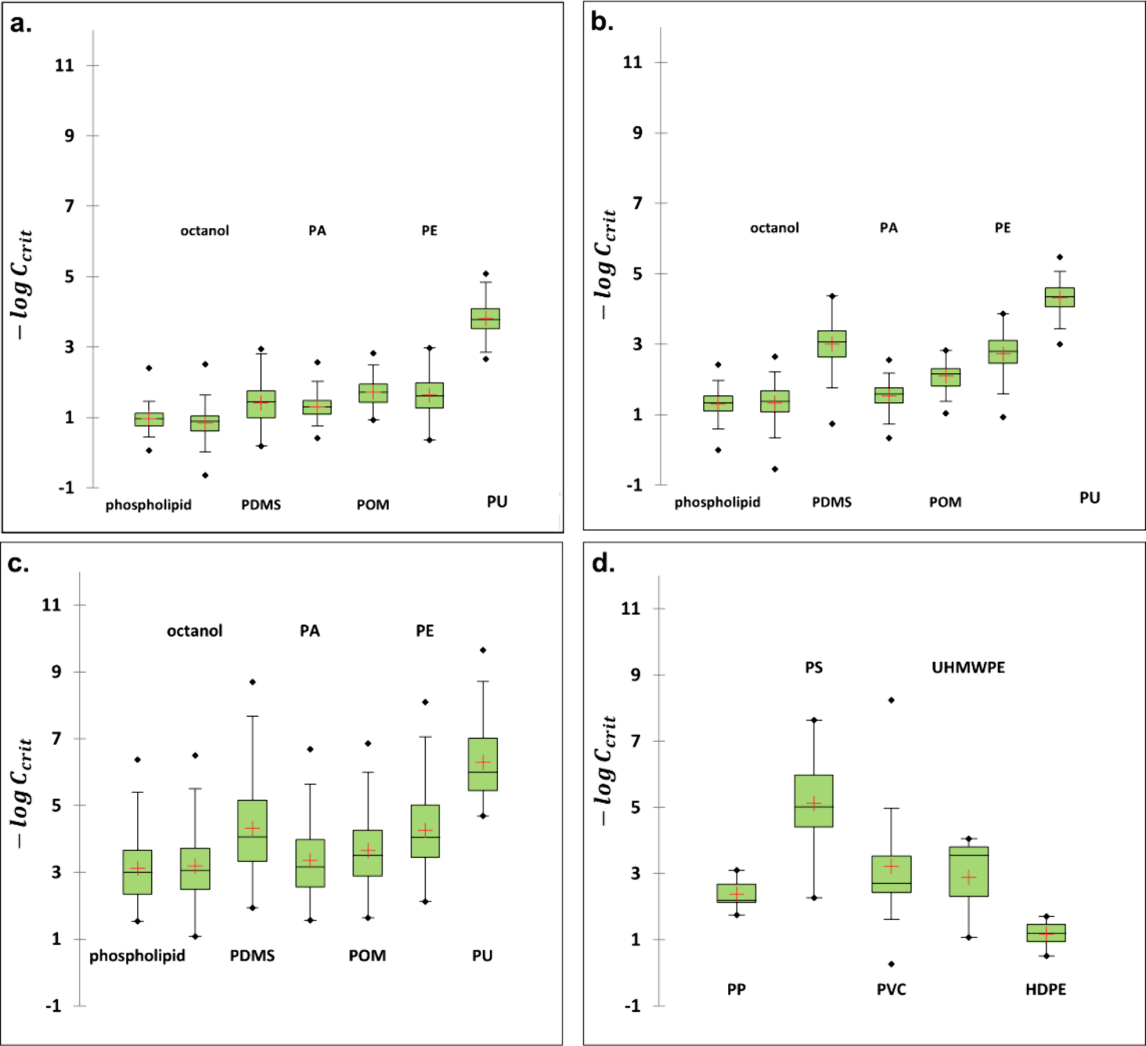
Distribution of critical burdens for various toxicants on the lipid, octanol, and plastic phases. Boxplots are shown for (a) baseline toxicants, (b) less inert toxicants, and (c) reactive toxicants, on PDMS, PA, POM, PE, and PU. Panel d shows the distributions for other plastic phases (PP, PS, PVC, UHMWPE, and HDPE), for which evaluation data were limited. The red symbols and the black horizontal lines within the boxes indicate the mean and median values of the critical burden distributions, respectively.

The critical burden of the baseline toxicants (*n* = 115) on phospholipid was calculated using eq 2, resulting in a value of 108.5 mmol (−0.96 log unit). This value is close to the critical burden calculated for octanol (−0.88 log unit) using the same group of chemicals. However, the critical burden values for octanol were found to have a more dispersed distribution compared with those observed for phospholipid (Figure 2). Overall, the calculated critical burden values for both phospholipid and octanol fall within the range of literature-reported values.^34,35^ The critical plastic burden is lower than the critical lipid and octanol burden, and it varies from 0.17 to 51.33 mmol for five different types of plastics, as shown in Figure 2a. Among five plastic types, PU has the lowest critical burden value, more than 2 orders of magnitude lower than that of phospholipid and octanol phases. On the other hand, PA exhibits the highest critical plastic burden value. These findings suggest that PA, with its relatively higher partition coefficient, can detect and quantify lower concentrations of contaminants in water more effectively compared to PU, which has a relatively lower partition coefficient and critical burden.

As anticipated, the critical lipid and plastic burden of the polar narcotics (*n* = 73) was found to be lower than that of nonpolar narcotics. This is attributed to the higher toxicity of polar narcotics compared to that of nonpolar narcotics. Specifically, the critical burden of polar narcotics on phospholipid (46.3 mmol/kg) and octanol (41.7 mmol/kg) was approximately half of the critical burden observed for nonpolar narcotics on these phases. Moreover, the critical plastic burden for the five types of plastics ranges from 0.04 to 6.90 mmol/kg, which is smaller than the critical lipid burden for nonpolar narcotics. Notably, PU was identified as the most sensitive plastic, requiring only a burden of 0.04 mmol/kg of PU to correspond to the median lethal concentration in the water phase. Conversely, PA exhibited the least sensitivity as a plastic phase, with a critical burden of 26.6 mmol/kg of PA.

Reactive toxicants are known to be highly toxic, and this is reflected in their critical burdens for the lipid and plastic phases. The difference in critical burden values between reactive toxicants and nonpolar narcotics is significant, with a difference of approximately 2 orders of magnitude observed for the lipid and plastic phases, except for PU. The PU phase, in particular, shows a much larger sensitivity to reactive toxicants. Conversely, the PA phase is the least sensitive plastic phase toward reactive toxicants, with a critical burden value of 6.8 × 10^–4^ mmol/kg.

The comparison of critical burdens for biotic and plastic phases estimated by the median and intercept methods is presented here. The intercept values are summarized in Table 1. The median values are depicted in Figure 2. For baseline toxicants, the critical burden values for the phospholipid and octanol phases were comparable between the two methods. Furthermore, the median method produced critical burden values for PA, POM, and PU that were in good agreement with those obtained by the intercept method (Table 1). However, differences of up to 0.50 log units were observed for the PDMS and PE phases when comparing the two methods. For less-inert chemicals, there were significant differences of more than 1 order of magnitude between the two methods for the critical octanol and lipid burdens. On the other hand, the differences between the two methods for the plastic phases except PE were not as large as those found for the lipid. For reactive toxicants, the critical burdens obtained from the two methods were similar in magnitude for these phases, except for the PU phase, which showed a difference of 1.6 log units.

These results show that while the median and intercept methods produce similar results for some phases and chemicals, there may be discrepancies for others. Overall, which method is more effective can be ascertained further by putting the values of critical burdens from both methods into the TLM and TPM to predict LC_50_ and comparing these predictions with the experimental values. This comparative test was performed, and the results are presented in the next section of the paper.

### 3.3 Prediction of Acute Toxicity from Critical Plastic Burden

**Figure 3:**
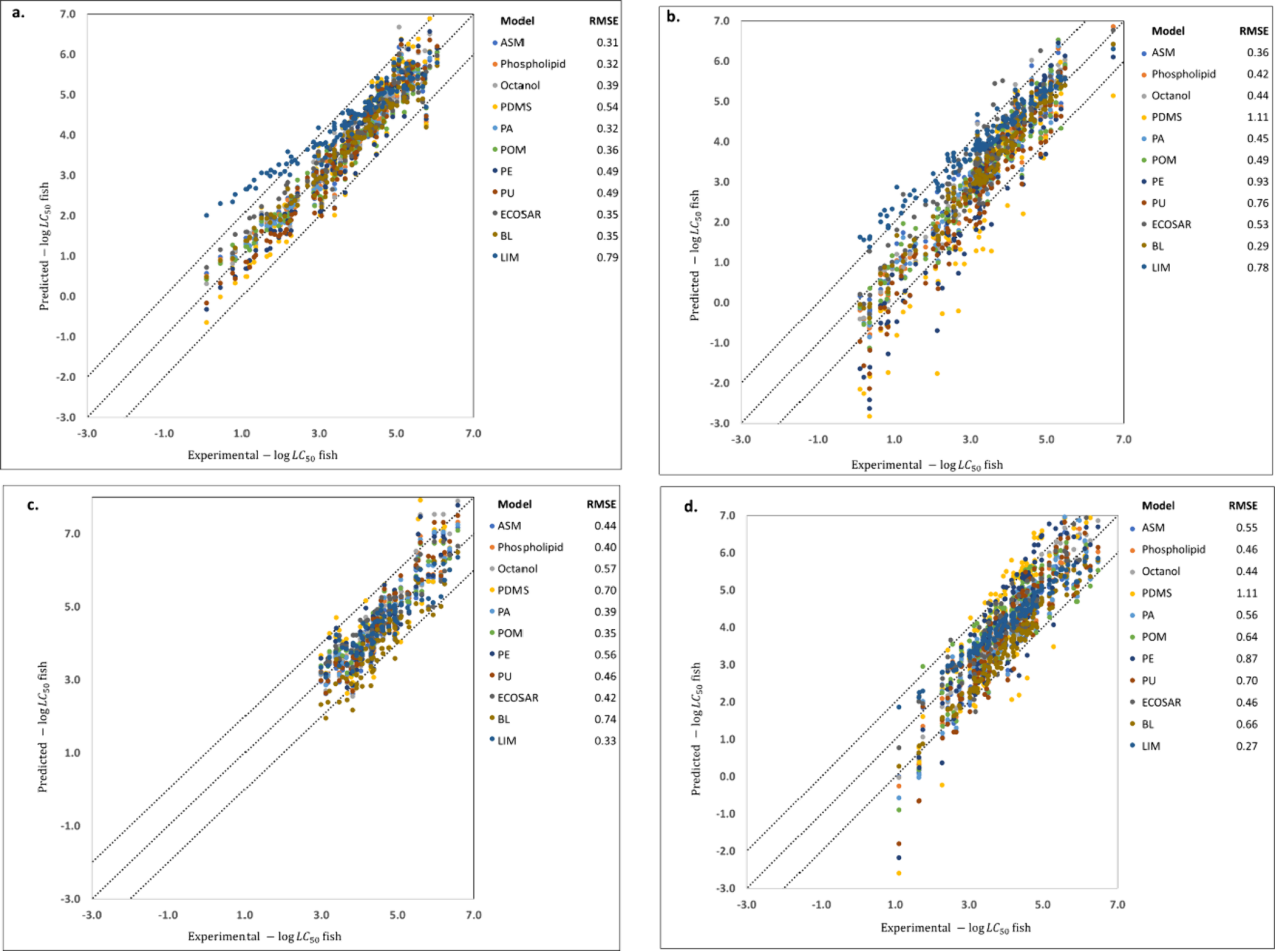
Comparison of LC_50_ predictions by the Target Plastic Model based on five plastic types (PDMS, PA, POM, PE, and PU) with experimental LC_50_ values for fish. The figure also displays predictions from other models, such as the Target Lipid Model based on phospholipid and octanol, as well as ASM, ECOSAR, BL, and LIM. Panels a and c present model evaluation using evaluation sets of baseline toxicants (*n* = 115) and less-inert toxicants (*n* = 73), respectively. Panels b and d show model validation using validation sets of baseline toxicants (*n* = 132) and less-inert toxicants (*n* = 128), respectively. RMSE values in log units are provided for each model in each panel, obtained by comparing predicted LC_50_ values with experimental values. The dotted line in the middle of each panel represents 1:1 agreement, while the upper and lower dotted lines indicate 1:2 agreement between the experimental and predicted LC_50_ values. For better readability, readers are encouraged to zoom in on the figure.

The critical burdens for the three groups of chemicals were estimated by using three different methods, and their effects on the accuracy of LC_50_ predictions were evaluated. For the baseline toxicants (*n* = 115), the TLM showed good agreement between experimental and predicted values of LC_50_ (Figure 3a). The input of critical burden values using three different methods into the TLM did not significantly affect the accuracy of the model (Figures 3a, S1, and S2). As expected, the TLM based on phospholipid performed better than the TLM based on octanol, as phospholipids are better representatives of membrane lipids.

The target plastic model exhibited a close agreement between its predicted values and experimental values for the five types of plastics, except for PU, which showed systematic deviations of 2.84 log units from the experimental values when using the 100 mmol method of critical burden. However, for the median and intercept methods, PU also demonstrated good agreement between predicted and experimental values (RMSE = 0.42 log unit). Overall, the input of critical burden values estimated using the median method into the TPM performed better than that of the 100 mmol and intercept methods. Thus, the median method of estimating the critical burden is recommended for input to the TPM.

Among plastic phases, the target PA and POM models performed the best with the lowest RMSE values of 0.311 and 0.343 log units for the median method, respectively. The prediction accuracy was on par with that of the target phospholipid model. The performance of the target PE model and target PU model was similar to that of the target octanol model. The target PDMS model exhibited RMSE = 0.538 log units when its predictions were compared to the experimental values.

The performance of the TPM was also compared with other models such as ASM, ECOSAR, BL, and LIM using the Baseline Evaluation Set (*n* = 115). These models exhibited RMSE values ranging from 0.349 to 0.306 log units (Figure 3a). The performance of these models was similar to the TLM and TPM. It should be noted that the experimental data set utilized to evaluate the TLM and TPM had already been employed to train regression models such as the BL and LIM. When comparing the predictions of two models against experimental data using the same training data, the model that has been trained on that data is expected to perform better than the nonfitted model, such as the TLM and TPM.

The Baseline Validation Set comprising 132 chemicals was used to evaluate the performance of the TPM compared to the TLM, ASM, ECOSAR, BL, and LIM (Figure 3b). The chemicals in the validation set were predicted to follow a baseline mode of toxic action, as shown by the toxic ratio or residual (experimental LC_50_ minus predicted LC_50_ by the baseline regression model) values < 1 log unit. Unlike the test set of 115 chemicals, the validation set was not used to compute the critical burdens for plastic, lipid, and octanol using median and intercept methods, thereby providing an unbiased evaluation.

The target phospholipid and octanol models showed good agreement between experimental and predicted values, with RMSE values of 0.42 and 0.44 log units, respectively (Figure 3b). The performance of the target PA model and POM model was similar to that of the target phospholipid and octanol models, with RMSE values of 0.45 and 0.49 log units, respectively. However, the target PU model and target PE model exhibited higher RMSE values of 0.76 and 0.93 log units, respectively. The poorest performing model was the target PDMS model with an RMSE value of 1.11 log units (Figure 3b).

In comparison, the ASM and ECOSAR models had RMSE values of 0.42 and 0.53 log units, respectively, for the Baseline Validation Set. Notably, predicted LC_50_ values obtained by the Target PDMS and Target PE Models for chemicals belonging to various classes, such as halogenated alcohols, diols, α,β-unsaturated alcohols, alcohol-ethers, diol-ethers, amines, amides, sulfoxides, and benzoic acids, differed from the experimental values by more than 1 order of magnitude. Many of these chemicals are hydrophilic and polar in nature. To investigate the performance of the models for chemicals with log *K*_ow_ >3, a subset of the validation set with 47 chemicals was analyzed. The results showed that the performance of the PDMS, PE, and PU models was improved by 0.48, 0.31, and 0.24 log units for this subset.

The predictive performance of TLM and TPM for polar narcotics is slightly inferior to that of nonpolar narcotics (Figure 3c). Other models, such as ASM, ECOSAR, and BL, also demonstrated relatively poor performance. This was expected because polar narcotics or less-inert toxicants may have specific interactions with the target organ that are not adequately represented by partitioning processes. The target phospholipid model and target octanol model have RMSE values of 0.40 and 0.57 log units, respectively. The target phospholipid model is more accurate than the target octanol model because it better represents the membrane lipids. The target plastic model has an RMSE range of 0.347–0.697 log units, with the POM being the most accurate and the PDMS being the least accurate. The LIM outperforms all other models with an RMSE of 0.335. However, it should be noted that unlike TLM and TPM, LIM is a fitted model trained on the same data set used for comparison, favoring its performance.

The Less-Inert Validation Set of 128 chemicals was used to validate the TPM for polar narcotics. These chemicals were predicted to follow a less inert mode of toxic action based on the predictions of LIM. Like the Baseline Validation Set, the chemicals in the Less-Inert Validation Set were not used to calculate the critical plastic, lipid, and octanol burdens, which helped ensure an unbiased evaluation of the models. The results showed that the predictions of the target phospholipid model and target octanol model were in good agreement with the experimental values for the 128 chemicals, with RMSE values of 0.46 and 0.44 log unit, respectively (Figure 3d). However, the TPM exhibited a wider range of RMSE values, ranging from 0.56 to 1.11 log unit, with PA performing the best and PDMS performing the worst when compared to the experimental values of LC_50_. In comparison, ASM and ECOSAR had RMSE values of 0.55 and 0.46 log units, respectively, for the same set of chemicals. Furthermore, the residuals for chemicals belonging to classes such as unsaturated alkenes, amine-alcohols, halogenated nitrobenzenes, and nitrogen-containing biphenyls were significantly higher for the PDMS and PE plastics.

Finally, the performance of various models, including the TLM, TPM, ASM, ECOSAR, BL, and LIM, were evaluated for reactive toxicants (*n* = 75). However, none of these models performed well for these chemicals (Figure S3). When the predicted values from these models were compared with experimental values for 75 reactive chemicals, the resulting RMSE values were all over 1 log unit. This poor performance was anticipated, since reactive toxicities are influenced by specific interaction parameters that are not accounted for in any of the models studied.

The critical plastic burdens and corresponding LC50 predictions for other plastic materials, including PP, PS, PVC, UHMWPE, and HDPE, were evaluated using limited available data. These evaluations showed critical burdens ranging from 0.01 to 63.89 mmol/kg of plastic. The agreement with ASM-predicted LC_50_ values varied, with PP and HDPE showing relatively better agreement (RMSE of 0.45 and 0.38 log units, respectively) and PS and PVC showing significant deviations (RMSE of 1.52 and 1.67 log units, respectively). Given the limited sample size and reduced chemical diversity used for these assessments, the results should be interpreted with caution. Detailed results and discussions for these other plastics are provided in section S1 of the SI.

## 4 Limitations

The target plastic model developed in this study has several limitations that must be considered. First, the model is not applicable to ionizable or reactive chemicals because they can undergo chemical reactions or ionization that affect their behavior and distribution in plastic phases, which are not fully explainable through equilibrium partitioning theory. Second, the model does not account for nonpersistent chemicals that undergo metabolism or physical or chemical transformation, which can change over time, making it difficult to predict their behavior accurately in plastic phases. Third, the model only considers passive exposure to chemicals and disregards active exposure, which may limit its applicability in certain environmental settings. Finally, the model does not consider adsorption of chemicals on plastics, only their absorption (partitioning), which may limit its accuracy for some plastic materials, as adsorption could be a crucial mechanism for their behavior in the environment.

Similarly, TPM, which focuses on the classification of chemicals based on their potential for baseline toxicity,^38^ inherently overlooks the receptor-mediated effects. This limitation is particularly relevant for compounds that exhibit significant chronic toxicity through specific receptor interactions. Receptor-mediated toxicity involves the specific interaction of chemicals with cellular receptors, leading to a cascade of biological events that can result in chronic toxicity at concentrations much lower than those required for baseline narcosis. Many compounds, including those within our study, have been documented to interact with cellular receptors, such as hormone receptors, neurotransmitter receptors, and various enzyme systems. These interactions can lead to significant adverse effects, including endocrine disruption, neurotoxicity, and immunotoxicity, which are critical for risk assessors to consider.

In applying the TPM to environmental plastics, several caveats must be addressed to ensure the accuracy and relevance of risk assessments. First, the impact of environmental weathering and biofouling on the partitioning properties of plastics is not well understood. A significant limitation of the model stems from its reliance on plastic–water partition coefficients derived from laboratory experiments using unweathered plastics. Significant alterations to these parameters by weathering and environmental conditions could lead to substantial inaccuracies in the risk assessments. While some studies^56,57^ using passive samplers like PDMS and PE in both freshwater and marine environments suggest minimal impact from these factors over periods up to 400 days, the long-term effects extending over several years remain critically underexplored.

Second, the effectiveness of TPM hinges on the assumption of equilibrium partitioning. Deviations from this equilibrium state can significantly impact the model’s predictions, particularly for high molecular weight and hydrophobic compounds, which may not equilibrate as quickly as compound having lower molecular weight and hydrophobicity.^56,57^ The variable surface-volume ratio of plastic^58^ further complicates this issue, with microplastics reaching equilibrium faster than macroplastics due to their greater surface area relative to volume.

Addressing these uncertainties requires prioritized research comparing the partitioning properties of weathered plastics collected from the environment to those of pristine plastics. This comparison provides deeper insights into how environmental aging affects partitioning behavior, which is pivotal for accurate TPM applications. Additionally, determining the age of environmental plastics through methods such as carbonyl index measurements^59,60^ could provide valuable data to evaluate their equilibrium status. Correlation analysis of these weathering indices with partition and diffusion coefficients would be helpful in understanding the changes in plastic properties as a function of weathering time, and thus fine-tuning the TPM. For instance, polyethylene microplastics from regions like the North Pacific Subtropical Gyre, often over 18 months old,^60^ are likely at equilibrium with very hydrophobic micropollutants, suggesting that similar assessments could be applied more broadly to gauge equilibrium in various environmental contexts. In summary, given these limitations, TPM should be applied with caution, ensuring that interpretations of results are informed by an awareness of its constraints.

## 5 Implications and Outlook

The framework of TPM developed in this study has several potential benefits for environmental scientists, which are described below.

First, like TLM, TPM can be applied to estimate the toxic unit. The toxic unit (TU) is a convenient way of calculating the toxicity of mixtures if components of the mixtures follow the same mode of toxic action. It is defined as
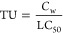
7

Eqs 4 and 5 can be rearranged in favor of *C*_w_ and LC_50_ for further insertion into eq 7 to obtain the following simplified form
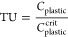
8

We can quantify the chemicals detected on environmental plastics or passive samplers and normalize these quantities to the critical plastic burden. This allows calculation of toxic units, which can be additives for baseline toxicants and used to determine mixture toxicity using eqs 9 and 10. As discussed above, environmental plastics might not always be at equilibrium due to varying conditions. These factors should be considered when interpreting chemical concentrations on environmental plastics. Despite these challenges, passive samplers provide a more controlled means of measuring bioavailable concentrations of contaminants, making them particularly useful for assessing baseline toxicity.
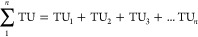
9
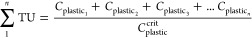
10

The sum of toxic units, ∑_1_^*n*^TU, can be transformed into the risk quotient, RQ, using an appropriate assessment factor (AF).
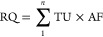
11

In the REACH framework,^61^ the typical assessment factor (AF) values are 1,000 for freshwater and 10,000 for marine conditions. These values are used to estimate the predicted no-effect concentration (PNEC) from acute toxicity data.^62^ The RQ via eq 11 may serve as an indicator, particularly as a preliminary assessment tool,^63^ to determine the potential risk to the water bodies.

Hence, this approach offers a significant advantage over existing methods for calculating mixture toxicity, as it does not rely on the availability of LC_50_ and plastic–water partition coefficient data, which can be limited, particularly for emerging chemicals. Only the knowledge of the critical plastic burden of a chemical, along with confirmation that its mode of toxic action is nonspecific narcosis, is required to calculate the toxic unit. For instance, while several hydrophobic micropollutants have been quantified in environmental plastic samples from Swiss surface waters,^64^ more insight could be gained from these studies by comparing the quantities with critical plastic burdens to calculate toxic units for risk assessment. However, as discussed earlier, factors such as equilibrium time, weathering, and environmental conditions must be considered when interpreting concentrations on environmental plastics. Given the success of TLM in predicting the toxicity of oil spills,^35^ TPM can be considered a promising method for estimating the toxicity of complex mixtures resulting from oil spills before and after weathering.

Second, the TPM holds promise as an animal-alternative technique for finding LC_50_ values for new chemicals in the laboratory. From the plastic phases considered in this study, it is clear that PA and POM are the most appropriate phases to be used as alternatives to fish in determining LC_50_ values for baseline toxicants. The target plastic model based on these plastic phases was able to predict the LC_50_ values for a wide range of chemicals within the range of experimental error. Consequently, with a consistent critical plastic burden, scientists would only need to measure the plastic–water partition coefficients for chemicals in PA and POM to reliably estimate LC_50_ values rather than conducting direct measurements with organisms. Future research should explore this potential application of the TPM, emphasizing its further development and validation.

Third, the TPM can be used in passive sampling-based field studies. Passive sampling is becoming increasingly popular among environmental scientists and regulatory authorities as it provides more insights into pollution risks by simultaneously determining the environmental levels and toxicities of detected chemicals. This approach can be particularly useful in situations such as marine oil spills with complex mixtures of hydrocarbons.

Finally, TPM may be a useful tool for designing passive-dosing-based toxicity experiments, as passive dosing techniques provide precise control over exposure concentrations. By utilizing the TPM, scientists can preselect appropriate passive doses that will encompass the critical plastic burden, leading to expected responses and resulting in a well-defined dose–response curve.

This study presents promising research avenues for the further development of a target plastic model. First, the model can be extended beyond fish to other aquatic species, following the success of the target lipid model for several aquatic species. Future studies could investigate the applicability of the target plastic model to a wider range of species. Specifically, incorporating species from at least two additional trophic levels below fish, such as algae and crustaceans, will fulfill the base-set data requirements as outlined in the REACH Guidelines.^61^ This expansion will enhance the model’s applicability and ensure a more comprehensive environmental risk assessment. Second, the target plastic model could be extended beyond acute toxicity to chronic toxicity levels. Similar to the target lipid model, the target plastic model can be applied to derive concentrations above which 95% of the species should be protected (HC_5_ values) for organic chemicals. These HC_5_ values can then be used to more accurately estimate PNECs, which could improve the accuracy and reliability of environmental risk assessments.^65^ Overall, these research avenues have the potential to enhance the utility of the target plastic model in environmental chemistry, providing new insights into the toxicity and behavior of organic chemicals associated with the plastic. Further research in these areas could lead to the development of more effective and efficient risk assessment methods, contributing to the protection of human and ecological health.

In summary, this study has shown that plastic phases exhibit behavior similar to biotic phases, allowing the development of a target plastic model based on the theoretical framework of the target lipid model. The target plastic model, specifically based on PA and POM plastic types, successfully predicted the acute toxicity end point for fish within the range of experimental errors. Environmental chemists can utilize the critical plastic burdens presented in this study for polar and nonpolar toxicants to rapidly estimate the toxicity of hundreds of thousands of chemicals associated with plastic.

## Data Availability

Data, including all molecular structures and their properties, are available in a machine-readable format as Supporting Information.

## Supporting Information Available

Information about the evaluation and validation sets used to develop the target plastic model and additional figures showing 1:1 comparison between the experimental and predicted values by Target Plastic Model with input method 1 and 3 of critical plastic burden(PDF)
A detailed Section on the Evaluation of the Target Model for Other Plastics (XLSX)
