# Supplementary material for: Assessing Aquatic Baseline Toxicity of Plastic-Associated Chemicals: Development and Validation of the Target Plastic Model
Source: J Chem Inf Model. 2024 Aug 9;64(16):6492–505. doi: 10.1021/acs.jcim.4c00574 (PMC11351055; doi:10.1021/acs.jcim.4c00574)
Supplement: Supplementary file 1 — ci4c00574_si_001.pdf [file ci4c00574_si_001.pdf]

Supporting Information File 1

Assessing Aquatic Baseline Toxicity of Plastic-Associated Chemicals: Development and Validation of the Target Plastic Model

Deedar Nabi[1,2], Aaron J. Beck[1], Eric P. Achterberg[1]

[1] GEOMAR Helmholtz Centre for Ocean Research Kiel

Wischhofstr. 1-3, 24148 Kiel, Germany

[2]Institute of Environmental Science and Engineering (IESE), School of Civil and Environmental Engineering (SCEE), National University of Sciences and Technology (NUST), H-12, Islamabad, Pakistan.

Section S1: Evaluation of Target Model for Other Plastics

The critical plastic burdens of chemicals on additional plastic types, such as polypropylene (PP), polystyrene (PS), polyvinyl chloride (PVC), ultra-high-molecular-weight polyethylene (UHMWPE), and high-density polyethylene (HDPE), were estimated tentatively. Due to the limited experimental data available for these other plastics, it was not possible to evaluate them in separate sets based on distinct modes of toxic action. As a result, the evaluation set for these plastic phases includes chemicals known to have different modes of toxic action. The values for the critical plastic burden on these plastic types fell within the range of 0.01 – 63.89 mmol/kg of plastic (Fig. 2d), with PS demonstrating the lowest and HDPE exhibiting the highest chemical critical burden. PVC demonstrated the highest variance in the distribution of critical burden, covering a range of more than seven orders of magnitude for 32 chemicals. This variance may be due to the fact that these chemicals belong to different chemical classes such as PCBs, PAHs, and

pharmaceuticals, which follow different modes of toxic actions. The critical burden of chemicals on HDPE (15.65 mmol/kg) was found to be similar to that on LDPE (24.95 mmol/kg), indicating that the density of polyethylene does not strongly affect the partitioning behavior of chemicals considered here.

It is important to note that the ASM estimated LC_{50} values were used to compute the critical burdens of chemicals on these plastic types due to a lack of experimental values. Additionally, the sample size and structural diversity of the chemicals used to evaluate these additional plastic types were limited compared to the plastic types used previously to evaluate and validate the target plastic model. Hence, the results for these additional plastic types should be interpreted with caution as they were based on estimated LC_{50} values due to the lack of experimental data and the limited sample size and structural diversity of chemicals for these plastic types.

The critical plastic burdens calculated for each plastic type were used to predict LC_{50} values using TPM. The predicted LC_{50} values were then compared to the ASM estimated values instead of experimental values due to the limited data available. The results showed a good agreement between the predicted LC_{50} values by the target PP model and by the ASM, with an RMSE of 0.45 log units. For the same set of chemicals (n=9), the target phospholipid and octanol models showed RMSEs of 0.11 and 0.17 log units, respectively. ECOSAR's predictions also matched well with ASM predictions, with an RMSE of 0.28 log units. However, the BL showed an RMSE of 0.61 log units with respect to the ASM predicted values for these chemicals. In this comparison, PCB 187 and 128 showed the highest residuals for the target PP model, which may be attributed to the poor data quality of measured partition coefficients of these strongly hydrophobic chemicals between PP and the water phase.

LC_{50} values predicted by the target PS model did not match favorably with the ASM predicted LC_{50} values based on 8 chemicals, showing an RMSE of 1.52 log units. In particular, nonylphenol showed a deviation of more than 2.75 log units from the ASM predicted value. Interestingly, the ECOSAR class for this chemical

is phenols, which generally exhibit excess toxicity compared to the baseline toxicity of neutral organics. Additionally, significant deviations between the predictions of the two models were observed for very hydrophobic PCB congeners such as PCB 171, 200, and 206. Although other models worked well for these chemicals, the deviation for the TPM may be attributed to the poor quality of the reported experimental plastic-water partition coefficients or to factors other than partitioning responsible for the exchange of chemicals between the polystyrene and water phases.

The performance of the target PVC model and ASM in predicting LC_{50} values for compounds from various chemical families was evaluated. Overall, the comparison of the predictions showed that the agreement between the models was not favorable, with an RMSE of 1.67 log units. However, this comparison included strongly hydrophobic chemicals such as PCB congeners and DEHP (di-2-ethylhexyl phthalate), which showed the highest deviations, not only for the target PVC model but also for other models used in this study. In contrast, the target PVC model's performance was satisfactory for moderately hydrophobic PCB congeners. For DEHP, even the ASM failed, as the difference between the experimental and ASM-predicted LC_{50} values was more than five orders of magnitude. For PCB 209, with a $\log K_{ow}$ of 8.27, the deviations for the PVC model were also more than five orders of magnitude. For most of the pharmaceutical drugs, there was good agreement between the predicted LC_{50} values of the PVC model and ASM. However, there were exceptions, such as flunitrazepam and chlorpromazine, which showed significantly higher residuals among the pharmaceutical drugs. Huge deviations between the predictions of two models for certain chemicals may be rationalized by considering specific toxic nature of the chemicals and/or the uncertain data quality stemming from the experimental challenges of measuring such chemicals in partitioning and toxicity experiments. For example, the ECOSAR class identified for DEHP is esters, implying it might be following a mode of toxic action other than baseline toxicity, and with a $\log K_{ow}$ value of 7.6, it also belongs to the strongly hydrophobic category. Measuring physicochemical properties of strongly hydrophobic chemicals free from experimental artifacts is a challenging task.

Similarly, flunitrazepam and chlorpromazine follow a mode of toxic action other than baseline, as indicated by their ECOSAR classes as amides and aliphatic amines, respectively. Such specific toxic modes of actions are difficult to account for by partitioning processes alone.

The performance of the target HDPE model in predicting LC_{50} values for chemicals was also evaluated. The comparison of the predictions revealed that the predicted LC_{50} by target HDPE model compared favorably with the ASM-predicted LC_{50}, as shown by its RMSE of 0.38 log units. Other models also performed well for this chemical set. However, the dataset used for this evaluation comprised only hydrocarbons with moderate hydrophobicities ($\log K_{ow}$ ranging from 2.73-5.81). These chemicals are known to follow the baseline toxic mode of action, and their measured partition coefficient values between the plastic and water phases are expected to not suffer too much from experimental artifacts. Therefore, the better performance of HDPE for such chemicals is not surprising. It is worth noting that the dataset used to evaluate the UHMWPE was not quite meaningful, as it only comprised three chemicals, with two belonging to the strongly hydrophobic category and one belonging to a mode of toxic action other than baseline toxicity.
